# Branched-chain amino acids and insulin resistance in type 2 diabetes: from metabolic dysregulation to therapeutic targets

**DOI:** 10.3389/fendo.2025.1643231

**Published:** 2026-02-02

**Authors:** Jie Mei, Fu-yuan Yang, Quan Gong

**Affiliations:** 1 Department of Anesthesiology, The First Affiliated Hospital of Yangtze University, Jingzhou, Hubei, China; 2 School of Basic Medicine, Yangtze University Health Science Center, Jingzhou, Hubei, China

**Keywords:** BCAAs, BCAAs metabolism, insulin resistance, mTOR signaling pathway, T2DM

## Abstract

Branched-chain amino acids (BCAAs) are a class of amino acids characterized by a branched aliphatic side chain, and they play critical physiological roles in humans, including protein synthesis, metabolic regulation, and immune system maintenance. Beyond serving as fundamental building blocks for protein biosynthesis, BCAAs and their metabolites also function as signaling molecules that regulate a variety of physiological processes, notably insulin secretion. Accumulating evidence indicates that plasma BCAAs levels are markedly elevated in patients with type 2 diabetes (T2DM), a phenomenon that may result from impaired activity of key enzymes in the BCAAs catabolic pathway, leading to metabolic dysregulation. It is widely recognized that BCAAs can activate the mTOR signaling cascade, thereby affecting insulin receptor sensitivity. In addition, aberrant BCAAs metabolism has been closely linked to alterations in the gut microbiota, which may further aggravate insulin resistance (IR). Taken together, dysregulated BCAAs metabolism may represent a critical mechanism underlying IR in T2DM. Therefore, this review summarizes current knowledge on BCAAs metabolism, explores its potential roles in the pathogenesis of IR in T2DM, and highlights emerging therapeutic strategies to reduce IR by targeting BCAAs metabolism.

## 1 Introduction

Type 2 diabetes mellitus (T2DM) is a metabolic disorder characterized by chronic hyperglycemia, primarily caused by insulin resistance (IR) and pancreatic β-cell dysfunction (1). IR is the state in which target cells exhibit reduced sensitivity to insulin, resulting in impaired glucose uptake and subsequent hyperglycemia (2). In parallel with the escalating obesity epidemic, the incidence of T2DM has risen steadily and is now regarded as one of the most pressing global public health issues. Data from the World Health Organization (WHO) indicate that as of 2022, an estimated 828 million individuals aged 18 years and older were diagnosed with diabetes, with T2DM accounting for the vast majority of cases (3). The prevalence of T2DM has risen most strikingly in developed countries and emerging economies, creating profound impacts on personal health and placing considerable strain on families and healthcare systems.

At present, management of T2DM mainly involves drug therapy, nutritional and physical activity interventions. Drug-based therapies primarily include insulin sensitizers, DPP-4 inhibitors, and SGLT2 inhibitors, all of which improve insulin efficacy and facilitate glycemic control (4–6). Nutritional interventions focus on controlling caloric and carbohydrate intake, aiming to manage body weight and enhance insulin responsiveness (7, 8). Physical activity is strongly advocated because it stimulates fat metabolism and potentiates insulin function (9). Nevertheless, despite these treatment options, a substantial proportion of patients fail to attain adequate blood glucose control, and the risk of diabetes-associated complications continues to increase over time. Consequently, discovering new biomarkers and therapeutic targets, especially those involved in metabolic pathways, has emerged as a major area of contemporary investigation.

Branched-chain amino acids (BCAAs) have recently attracted increasing attention as a class of biologically important amino acids. Comprising leucine, isoleucine, and valine, BCAAs are essential amino acids acquired from the diet, involved in intracellular protein synthesis and energy homeostasis. A growing body of research demonstrates that BCAAs significantly contribute to diabetic metabolism, closely linking them to the development and progression of IR. In individuals with T2DM, circulating BCAAs concentrations are generally higher than in healthy populations, correlating strongly with IR, obesity, and other metabolic abnormalities (10). Experimental data indicate that BCAAs function beyond metabolic intermediates, potentially influencing insulin responsiveness via diverse mechanisms and aggravating IR. The interplay between BCAAs and IR involves intricate regulatory mechanisms. BCAAs engage in cellular growth and metabolic control through mTOR signaling, with hyperactivation of mTOR being strongly associated with IR (11). Moreover, metabolites derived from BCAAs, including α-keto acids and ketone bodies, may compromise β-cell functionality, resulting in diminished insulin output. In recent years, research has increasingly focused on the gut microbiota’s involvement in BCAAs metabolism. Evidence suggests that imbalances in gut microbial composition can modulate BCAAs uptake and metabolic processing, indirectly impacting insulin signaling (12).

Although the reasons for elevated BCAAs levels and their association with IR remain unclear, dysfunctional BCAAs catabolism may represent one of the underlying factors. This review aims to provide an in-depth understanding of BCAAs metabolism and its potential role in the pathogenesis of IR in T2DM, as well as to summarize pharmacological and alternative lifestyle interventions that reduce plasma BCAAs levels and their impact on metabolic health.

## 2 The relationship between IR and amino acid metabolism

IR was initially characterized in Himsworth’s seminal studies (13). Early studies demonstrated that diabetic individuals showed divergent responses to glucose and insulin administration, with some maintaining stable or declining blood glucose and thus categorized as insulin-sensitive; whereas others exhibited marked hyperglycemia, reflecting insulin insensitivity or attenuated insulin responsiveness. This phenomenon typifies IR in metabolic syndrome, characterized by the inability of peripheral tissues to elicit an appropriate glucose-lowering response at normal circulating insulin concentrations, commonly termed reduced insulin sensitivity. Such insulin action encompasses the inhibition of hepatic glucose production, suppression of lipolysis, stimulation of glucose uptake, and augmentation of glycogen synthesis (14–18). IR predominantly impacts skeletal muscle, hepatic tissue, and white adipose depots (19). IR is commonly linked to impairments across multiple insulin signaling cascades, involving ectopic lipid deposition, mitochondrial dysfunction, and enhanced activation of stress-related kinases such as c-Jun N-terminal kinase (JNK) and pro-inflammatory pathways (20).

In recent years, dysregulation of BCAAs metabolism has received increasing attention in obesity-related disorders, including IR, T2D and cardiovascular diseases. A plasma metabolomic analysis comparing obese and lean individuals revealed significant differences in BCAAs-related metabolites, suggesting that enhanced BCAAs catabolism is associated with IR. This finding has been further validated in mouse plasma metabolomic studies (21–23). Moreover, in a prospective cohort study, targeted metabolomic analysis demonstrated that elevated levels of several amino acids, including BCAAs, were significantly associated with an increased risk of developing T2D (24). These findings indicate that BCAAs may serve as both biomarkers and key mediators in obesity and related metabolic disorders. In addition, a strong correlation has been observed between circulating BCAA levels and IR (25, 26). Furthermore, high BCAAs concentrations have been shown to impair insulin sensitivity and induce IR. In animal studies, acute elevation of circulating BCAAs led to increased blood glucose and insulin levels and significantly reduced whole-body insulin sensitivity during hyperinsulinemic–euglycemic clamp experiments, whereas lowering BCAAs levels improved glucose tolerance in obese mice (27). A randomized, double-blind study investigated the effect of modulating BCAAs metabolism on insulin sensitivity in 16 patients with mild to moderate T2D using sodium phenylbutyrate (NaPB), a drug that promotes BCAAs catabolism. The results showed that NaPB treatment significantly reduced plasma BCAAs levels and improved peripheral insulin sensitivity by approximately 27%, accompanied by enhanced skeletal muscle mitochondrial oxidative capacity, increased glucose oxidation, and decreased blood glucose levels (28).

The two major mechanisms by which plasma BCAAs are related to IR are as follows: First, impaired mitochondrial BCAAs metabolism plays a critical role (29). Patients with T2DM exhibit higher plasma BCAAs levels compared to healthy individuals, while their mitochondrial oxidative capacity is markedly reduced (30). Elevated plasma BCAAs may result from diminished mitochondrial oxidative capacity, suggesting a close link between mitochondrial function and IR (31). When BCAAs metabolism is impaired, large amounts of BCAAs -derived metabolites (such as 3-hydroxyisobutyrate, 3-HIB) accumulate in plasma, which may exert toxic effects on cells and ultimately lead to IR (32). Reportedly, a community-based observational study including 15 patients with T2DM, 13 first-degree relatives (FDR), and 17 controls (CON, all overweight or obese) assessed muscle mitochondrial oxidative capacity using high-resolution respirometry, and in some participants combined hyperinsulinemic-euglycemic clamp with ¹³C-leucine tracer to measure *in vivo* BCAAs oxidation. Results showed that plasma BCAAs levels in T2DM patients were higher than in controls and were significantly negatively correlated with muscle mitochondrial oxidative capacity (r = -0.44, P < 0.001). Furthermore, their whole-body leucine oxidation rate was significantly reduced under basal conditions (0.202 vs. 0.275 μmol·kg^−1^;·min^−1^, P < 0.05), and also showed a downward trend under hyperinsulinemic conditions (30).

The second mechanism involves activation of the mTOR signaling pathway by BCAAs metabolism, thereby disrupting insulin signaling (33). In addition to their role in protein synthesis, BCAAs act as signaling molecules regulating multiple physiological activities (34). Studies have shown that plasma leucine can influence glucose metabolism by activating the mTOR signaling pathway in skeletal muscle. Linn et al. reported that during hyperinsulinemic-euglycemic clamp experiments, ingestion of whey protein or an equivalent amount of leucine led to a significant ~30% increase in muscle p-mTOR (Ser2448) levels (35). Conversely, studies with the mTOR inhibitor rapamycin provide supporting evidence from the opposite perspective: in patients with type 1 diabetes, short-term rapamycin pretreatment prior to islet transplantation significantly reduced daily insulin requirements (–8 ± 6 U/day, p < 0.001) and led to sustained improvement in hepatic insulin sensitivity one year post-transplant, as indicated by a significant decrease in hepatic glucose production (–1.1 ± 1.1 mg/kg/min, p = 0.04) (36). In addition, insulin can activate mTOR through the PI3K-Akt signaling pathway (37). When BCAAs continuously activate mTOR, the downstream effector S6 kinase (p70S6K) phosphorylates insulin receptor substrate 1 (IRS-1) on serine residues, thereby further inhibiting Akt signaling, reducing glucose transport, and promoting the development of IR (38, 39).

Therefore, BCAAs are not only metabolic biomarkers of IR but may also serve as one of the key drivers in the development of IR.

## 3 BCAAs metabolism and regulation

### 3.1 BCAAs metabolic pathways

**Figure 1:**
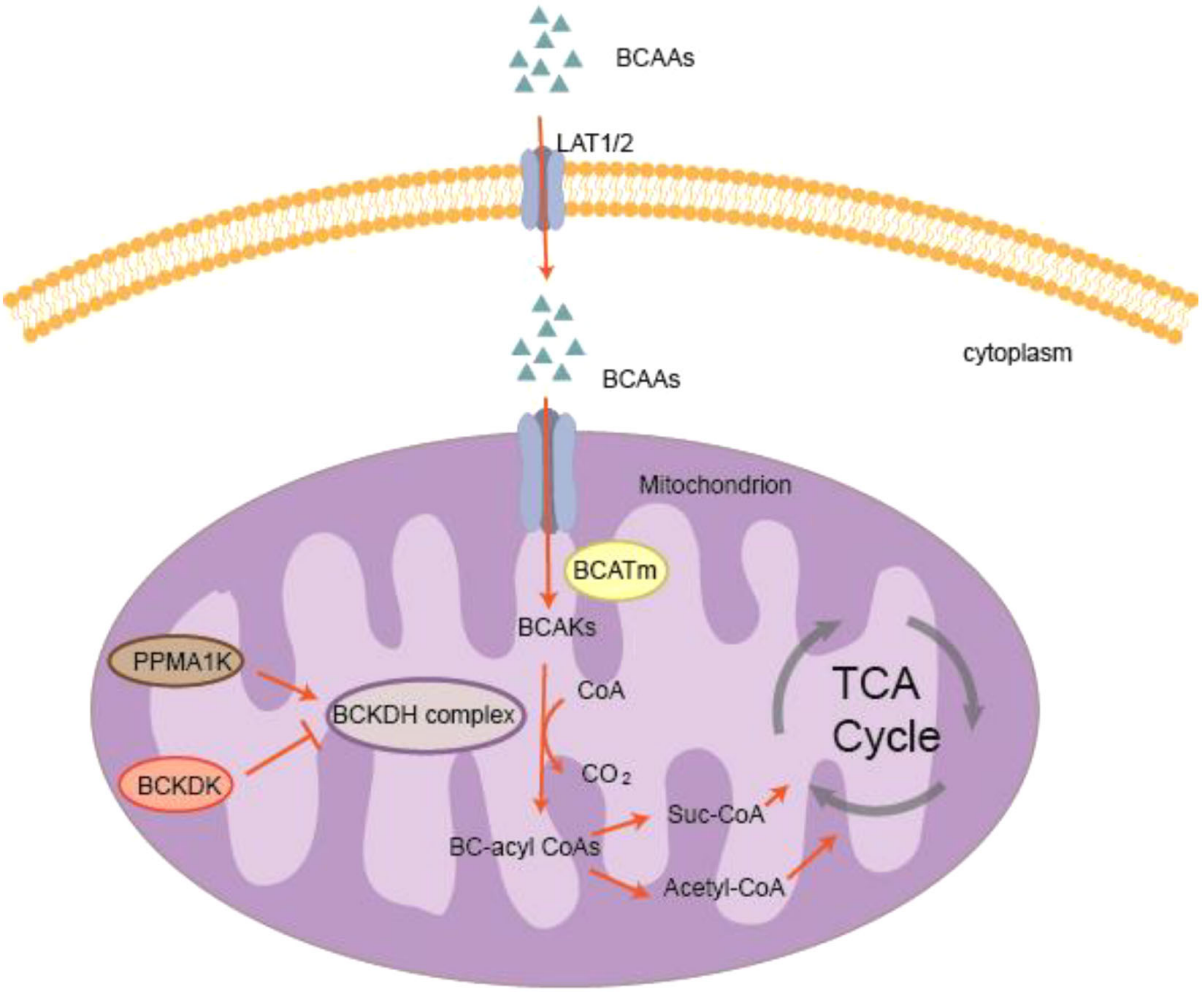
Overview of the BCAA catabolic pathway. BCAAs are transported across the cell membrane into the mitochondria where they are converted to BCKAs by branched-chain amino acid transaminase (BCATm). BCKAs are then oxidized by the BCKDH complex to form branched-chain acyl-CoAs (BC-acyl-CoAs). BCKDH complex activity is regulated by BCKDK (inhibited by phosphorylation) and PPM1K (activated by dephosphorylation). The branched-chain acyl-CoAs are further converted to acetyl-CoA and succinyl-CoA, which enter the TCA cycle.

Plasma BCAAs mainly originate from three sources: dietary intake, tissue proteolysis, and gut microbial synthesis (40), among which dietary intake is the primary source. After ingestion, BCAAs are primarily absorbed in the intestine via peptide transporters (41), and subsequently transported into cells through multiple amino acid transporters, such as LAT1 (encoded by SLC7A5) and LAT2 (encoded by SLC7A8) (42) (see **Figure 1**). The first two steps of BCAAs metabolism are catalyzed by branched-chain aminotransferase (BCAT) and branched-chain α-keto acid dehydrogenase (BCKDH), respectively. BCAT consists of two isoenzymes, BCAT1 and BCAT2 (43). BCAT1 is a cytosolic enzyme distributed in tissues such as embryos, ovaries, and the brain, whereas the mitochondrial enzyme BCAT2 is expressed in most tissues, particularly in skeletal muscle and adipose tissue. The first step of BCAAs metabolism is a reversible transamination reaction catalyzed by BCAT, producing branched-chain α-keto acids (BCKAs). Specifically, leucine, isoleucine, and valine are converted to α-ketoisocaproate, α-keto-β-methylvalerate, and α-ketoisovalerate, respectively (44). The second step is an irreversible oxidative decarboxylation catalyzed by BCKDH complex (45), which converts BCKAs into various branched-chain acyl-CoA derivatives. The BCKDH complex is composed of three catalytic subunits: α-keto acid dehydrogenase (E1 component), dihydrolipoyl transacylase (E2 component), and dihydrolipoyl dehydrogenase (E3 component). Its activity is tightly regulated by phosphorylation and dephosphorylation (46). Under the action of BCKDH complex, the CoA derivatives generated from BCKAs are further metabolized into acetyl-CoA and succinyl-CoA, which subsequently enter the tricarboxylic acid (TCA) cycle (47).

### 3.2 Factors affecting BCAAs metabolism

#### 3.2.1 Dietary intake

Diet is the primary determinant of plasma BCAAs levels. High-protein diets, particularly those rich in animal protein, can acutely elevate circulating BCAAs. A randomized double-blind crossover study involving 27 healthy adults demonstrated that postprandial BCAAs metabolism is significantly influenced by dietary protein content. After consuming isocaloric high-protein (HP) and low-protein (LP) meals, plasma BCAAs and their catabolic products were monitored over 5 hours. Results showed that BCAAs, related keto acids, and acylcarnitines were significantly higher in the HP group than in the LP group, with the exception of the branched-chain α-keto acid α-ketoisovalerate, which exhibited higher postprandial concentrations following LP intake; however, when BCAAs levels exceeded a certain threshold, their catabolic products decreased (48). On the other hand, the effects of long-term diet may differ. A one-month intervention study involving 102 healthy adults found that, although dairy protein intake differed significantly between groups, fasting BCAAs levels and insulin sensitivity did not change significantly (49).

Notably, dietary composition influences not only the absolute levels of BCAAs but also their distribution within metabolic networks. For instance, the combined effect of high-fat diets and elevated BCAAs intake has been found to exacerbate obesity and IR. Moreover, certain micronutrients, such as thiamine (vitamin B1), may indirectly modulate BCAAs catabolism by regulating BCKDC activity (50).

#### 3.2.2 Gut microbiota

The gut microbiota is a critical regulator of host metabolism, playing central roles in immunity, metabolism, structural integrity, and neuroregulation (51–53). BCAAs, as essential amino acids and key components of proteins, are abundant in animal-based diets. Through their involvement in intestinal physiological processes, the gut microbiota influences host metabolism and overall health. In recent years, dysbiosis has been implicated in obesity and other metabolic disorders (54). Notably, patients with diabetes often exhibit an imbalance in the gut microbiota, characterized by an enrichment of BCAAs -producing bacteria (e.g., *Staphylococcus aureus, Prevotella copri*) (55, 56) and a depletion of BCAAs -degrading beneficial bacteria (e.g., *Faecalibacterium prausnitzii*) (57). This imbalance promotes the accumulation of circulating BCAAs, thereby aggravating IR. Studies have shown that interventions such as mulberry leaf extract and pyridostigmine can remodel the gut microbial composition, reduce BCAAs biosynthesis, and enhance BCAAs catabolism, ultimately improving insulin sensitivity (58, 59).

#### 3.2.3 Enzymatic molecules and regulation

As with many metabolic pathways, the regulation of BCAAs catabolism primarily occurs at its rate-limiting steps. In the first transamination reaction, BCAT2 (also known as mitochondrial branched-chain aminotransferase, BCATm) serves as the key enzyme, playing a critical role in the initial phase of BCAAs metabolism. Its deficiency leads to the accumulation of BCAAs and BCKAs (60). In a study of cardiac-specific BCATm knockout mice, researchers observed reduced cardiac BCAAs oxidation, elevated BCAAs levels, and decreased BCKA levels. Interestingly, insulin-stimulated glucose oxidation in the heart was enhanced, accompanied by increased Phosphorylated Akt (p-AKT), indicating improved insulin sensitivity. Further experiments demonstrated that high levels of BCKA infusion markedly suppressed insulin-stimulated glucose oxidation, reduced p-AKT, and inactivated pyruvate dehydrogenase (61). In the second oxidative decarboxylation step, the BCKDH complex acts as the rate-limiting enzyme. Defects in the BCKDH complex result in the accumulation of metabolic intermediates and mitochondrial dysfunction. Moreover, impaired BCKDH complex activity can disrupt BCKA metabolism, leading to their accumulation and perturbation of normal BCAAs catabolic pathways. For example, in patients with maple syrup urine disease, BCKDH complex activity is markedly reduced (62), and fibroblasts derived from these patients exhibit elevated levels of reactive oxygen species (ROS) (63). Such defects not only impair BCKA metabolism but may also interfere with insulin signaling and disrupt lipid metabolism, thereby contributing to the development of IR.

In addition, there are two key enzymes that play important roles in regulating the activity of the BCKDH complex. One of them is branched-chain α-keto acid dehydrogenase kinase (BDK, also known as BCKDK), whose phosphorylation inactivates the BCKDH complex, leading to BCAAs accumulation (64). Studies have shown that inhibition of BDK can promote metabolic benefits. For example, thiazole-based BDK inhibitors can improve heart failure and metabolic function in mice (65), while the small-molecule allosteric BDK inhibitor BT2 can effectively alleviate non-alcoholic fatty liver disease and multifactorial metabolic disorders in mice (66). The other enzyme that regulates the BCKDH complex is protein phosphatase mitochondrial 1K (PPM1K), which activates the complex through dephosphorylation (67). Research has found that female mice with PPM1K deficiency exhibit elevated BCAAs levels and associated metabolic disturbances (68); in humans, PPM1K deficiency can lead to BCKDH complex deficiency, resulting in mild maple syrup urine disease (69).

#### 3.2.4 Tissues, organs, and functions

BCAAs are not only essential substrates for protein synthesis but also play pivotal roles in energy metabolism and signal transduction. Their systemic levels are jointly determined by dietary intake, metabolic utilization, and regulation by multiple organs and tissues. Distinct organs exhibit tissue-specific functions in BCAAs metabolism and clearance, thereby profoundly influencing circulating BCAAs concentrations and metabolic health.

Skeletal muscle serves as the primary site for BCAAs catabolism, with the highest activities of branched-chain aminotransferase (BCAT) and BCKDH complex, followed by adipose tissue and the brain (47). Unlike most amino acids, which are primarily catabolized in the liver, BCAAs undergo limited initial metabolism in this organ. Instead, they are preferentially taken up by skeletal muscle, where they are transaminated by mitochondrial BCAT (BCATm) to generate BCKAs, which subsequently enter mitochondrial oxidative metabolism. Beyond serving as energy substrates, BCAAs in skeletal muscle also regulate insulin signaling and the mTOR pathway, making muscle mass and metabolic state critical determinants of BCAAs clearance efficiency (70). In obesity or IR, impaired muscle utilization of BCAAs leads to elevated circulating levels (71).

Adipose tissue also plays an important role in the regulation of BCAAs metabolism. Mammalian adipose tissue includes white and brown adipose tissues (WAT and BAT, respectively) (72). WAT is a robust energy‐storage and endocrine organ and is essential for maintaining metabolic health during aging (73). In multiple obesity models (fa/fa rats, db/db mice, and diet‐induced obese mice), the protein level of the branched‐chain α‐ketoacid dehydrogenase (BCKD) complex (E1α subunit) is significantly reduced in WAT. When insulin action is impaired and/or metabolic signaling is disrupted in WAT, BCAAs utilization by WAT may be compromised (74). BAT is not only a thermogenic organ but also an important “metabolic filter” that regulates BCAAs metabolism and maintains systemic metabolic health. It has been reported that under cold stimulation, BAT transports BCAAs into mitochondria via SLC25A44 for oxidation, thereby supporting thermogenesis and promoting whole‐body BCAAs clearance. Impaired BCAAs metabolism in BAT leads to reduced BCAAs clearance and limited thermogenic capacity, which in turn promotes high‐fat diet–induced obesity and glucose intolerance. In addition, studies have shown that differentiating adipocytes increase BCAAs utilization and release metabolic intermediates such as 3‐hydroxyisobutyrate (3‐HIB), which enhances fatty acid uptake and storage, thereby exacerbating obesity and IR (75). To some extent, WAT and BAT can interconvert. Acetyl‐CoA derived from branched‐chain keto acids (BCKAs) can acetylate PR domain‐containing protein 16 (PRDM16) at lysine 915 (K915), thereby suppressing WAT browning. In contrast, depletion of acetyl‐CoA markedly promotes WAT browning and enhances energy expenditure (76).

The brain critically depends on BCAAs homeostasis for normal function. The blood–brain barrier transporter SLC7A5 plays a central role in mediating BCAAs entry into the brain, and its dysfunction leads to altered brain amino acid composition, impaired protein synthesis, severe neurological abnormalities, and has been linked to autism spectrum disorders (77). Furthermore, BCAAs and aromatic amino acids (ArAAs) compete for transport into the brain. Elevated plasma BCAAs levels increase brain uptake of BCAAs while reducing ArAA availability, thereby suppressing the synthesis of neurotransmitters such as serotonin and catecholamines, ultimately affecting mood regulation, hormonal balance, and blood pressure control (78). Importantly, BCAAs serve as major nitrogen donors for glutamate synthesis in the brain, with approximately one-third of glutamate nitrogen derived from BCAAs, particularly leucine. Through the astrocyte–neuron “leucine–glutamate cycle,” nitrogen transfer and recycling are maintained, supporting neurotransmitter synthesis and buffering excessive glutamate to prevent excitotoxicity (79).

## 4 Effects of BCAAs on IR

BCAAs are not only important biomarkers of IR but may also directly contribute to its development. In an acute supplementation experiment, intravenous BCAAs infusion transiently reduced blood glucose in mice, likely through stimulation of insulin secretion. However, beginning 10 minutes post-infusion, plasma insulin and glucose levels both rose and remained elevated until the end of the experiment. Hyperinsulinemic–euglycemic clamp studies further demonstrated reduced systemic insulin sensitivity, accompanied by hyperactivation of hypothalamic AgRP neurons and increased food intake. These effects were reversed when BCAAs levels were reduced (27). Thus, BCAAs can acutely impair glucose metabolism and insulin sensitivity, while dietary restriction of BCAAs has shown beneficial effects in experimental models. In a protein diet study, reducing all three BCAAs by 67% in the control amino acid (Ctrl AA) diet (Low BCAAs) exerted distinct effects on obese mice. Specifically, isoleucine restriction reprogrammed hepatic and adipose metabolism, enhanced hepatic insulin sensitivity and ketogenesis, increased energy expenditure, and activated the FGF21–UCP1 axis. Valine restriction induced similar but milder metabolic benefits, whereas leucine restriction had no significant effect (80, 81).

Mechanistically, BCAAs contribute to IR through several pathways. First, excessive BCAAs and their metabolites (e.g., BCKAs and corresponding acylcarnitines) accumulate in skeletal muscle and liver, impairing mitochondrial energy metabolism and inhibiting fatty acid oxidation, leading to by-product accumulation and further disruption of insulin signaling. Second, BCAAs activate the mTORC1 pathway, inducing serine phosphorylation of IRS-1 and weakening downstream insulin receptor signaling, thereby reducing cellular insulin sensitivity. Third, elevated BCAAs levels promote M1 polarization of adipose tissue macrophages, increasing proinflammatory cytokines such as interleukin-1β (IL-1β), tumor necrosis factor-α (TNF-α), and monocyte chemoattractant protein-1 (MCP-1), which further exacerbate IR (82).

## 5 BCAAs and mTOR signaling pathway activation

Mammalian target of rapamycin (mTOR) is an evolutionarily conserved serine/threonine protein kinase and a key member of the phosphatidylinositol 3-kinase-related kinase (PIKK) family (83). mTOR regulates core processes of cell proliferation and growth in response to signals such as insulin, amino acids, energy status, and oxygen (84). It coordinates upstream signals with downstream effectors, including transcriptional and translational mechanisms, to modulate fundamental cellular activities such as energy utilization, protein synthesis, autophagy, cell growth, and proliferation (85). As a catalytic subunit, mTOR exists in two distinct multiprotein complexes: mTOR complex 1 (mTORC1) and mTOR complex 2 (mTORC2), which phosphorylate different substrates and exhibit diverse physiological functions (86).

**Figure 2:**
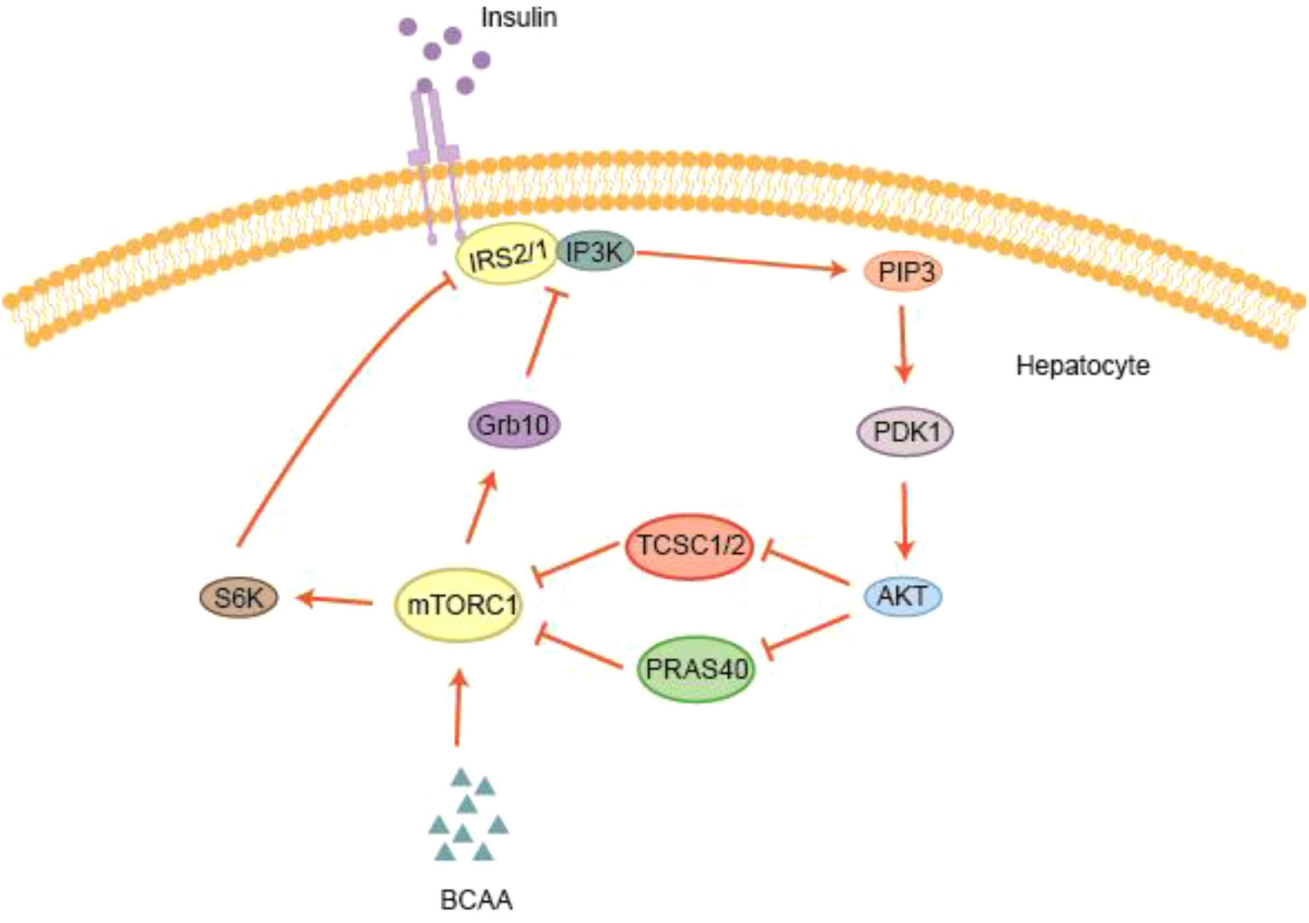
BCAA activation of the mTOR signaling pathway. BCAAs can stimulate mTORC1 in the PI3K/AKT signaling pathway, which activates downstream molecules such as S6K1 and Grb10. S6K1 and Grb10 mediate the degradation of IRS, thereby disrupting insulin signaling.

Patients with obesity and insulin-resistant T2DM exhibit significantly elevated serum BCAAs levels (87, 88). Clinical studies show that elevated BCAAs levels are positively correlated with the degree of IR (89). BCAAs, including leucine, isoleucine, and valine, are essential amino acids and potent activators of mTORC1 (90). Leucine can synergize with insulin to activate mTORC1. Persistent mTORC1 activation leads to phosphorylation of S6K1, which subsequently phosphorylates serine residues on insulin receptor substrates IRS-1 and IRS-2 impairing downstream insulin signaling and potentially targeting IRS-1 for proteasomal degradation (38, 39) (see **Figure 2**). For example, a study found that when rat extensor muscles were cultured with varying concentrations of leucine, AMP-activated protein kinase (AMPK) activity was suppressed, while the mTORC1/p70S6K1 signaling pathway was activated, resulting in IR (91).

Grb10 is an intracellular adaptor protein that can directly bind to multiple growth factor receptors, including the insulin receptor and the insulin-like growth factor-1 (IGF-1) receptor, and negatively regulate their actions (92). mTORC1 can stabilize Grb10 by phosphorylating its tyrosine residues, preventing its degradation and thereby continuously inhibiting the interaction between IRS and the insulin receptor. This leads to increased IR and places an additional burden on pancreatic glucose handling. Consequently, BCAAs may contribute to pancreatic dysfunction or the development of T2DM through sustained activation of mTORC1.

BCAAs, particularly leucine, directly act on downstream targets S6K1 and Grb10 via mTORC1 signaling. Activation of S6K1 promotes protein synthesis and cell growth, while Grb10 mediates negative feedback to suppress insulin signaling, helping maintain metabolic homeostasis. A deeper understanding of the roles of S6K1 and Grb10 in the mTORC1 pathway is critical for elucidating the contribution of BCAAs to T2DM-associated IR and provides potential targets for therapeutic strategies based on mTOR signaling.

## 6 Effects of increased BCAAs on insulin signaling disruption

**Figure 3:**
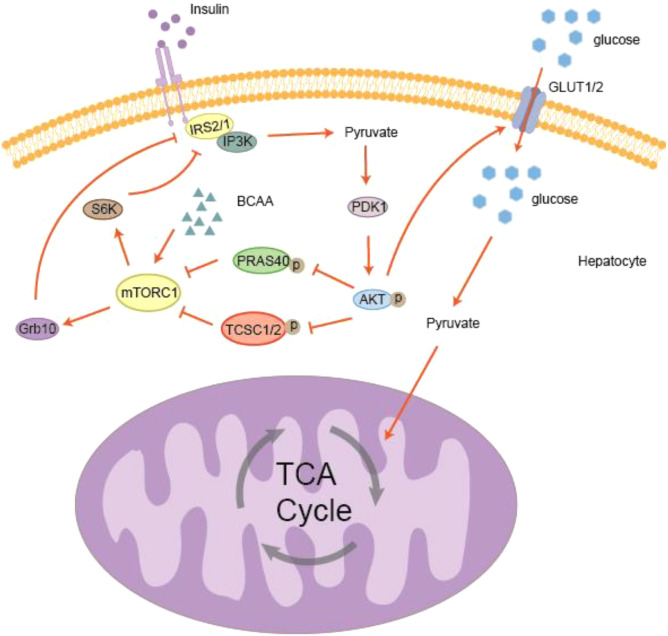
BCAA metabolism and insulin resistance. Elevated BCAA levels promote mTORC1 activation, which negatively regulates insulin signaling through phosphorylation of the downstream targets S6K and Grb10. First, PI3K activates AKT upon activation of the insulin receptor substrate (IRS). Activated AKT phosphorylates and inhibits TSC2, while phosphorylation of PRAS40 further enhances mTORC1 activity. Subsequently, mTORC1 activates S6K and Grb10. Increased S6K activity results in negative feedback phosphorylation of IRS, leading to IRS degradation and inhibition of insulin signaling. At the same time, Grb10 activation also suppresses IRS function. These mechanisms ultimately disrupt GLUT1/4 translocation, reducing glucose uptake and utilization, leading to insulin resistance.

Insulin promotes the translocation of GLUT4 in adipocytes through activation of the PI3K/AKT signaling pathway (see **Figure 3**), facilitating glucose uptake into fat cells. Intracellular glucose is metabolized via glycolysis to glycerol, which participates in triacylglycerol (TAG) synthesis and *de novo* lipogenesis (93). Adipocyte IR is an early hallmark of obesity and T2DM. BCAAs can impair insulin action through sustained activation of mTORC1, with leucine being a key activator. mTORC1 is involved in multiple physiological processes, including cell growth, metabolism, and glucose homeostasis (94). Studies have shown that in rats fed a high-fat (HF) diet supplemented with BCAAs, IR can be reversed by the mTORC1 inhibitor rapamycin (22). Additionally, the microbiota-dependent tryptophan metabolite 5-hydroxyindole-3-acetic acid (5-HIAA) can enhance hepatic insulin signaling by directly activating the aryl hydrocarbon receptor (AhR), stimulating TSC2 transcription, and suppressing mTORC1 signaling, thereby alleviating HF diet-induced IR (95).

In obese and diabetic rodents and humans, the downstream molecule of mTORC1, ribosomal S6K1, is activated (96). S6K1 is regarded as a negative regulator of IRS signaling (38, 39) (96–98). Experimental evidence indicates that high concentrations of BCAAs stimulate mTOR, subsequently inducing IR through serine phosphorylation of IRS-1 (99). In addition, dysregulated BCAAs metabolism can cause BCAAs accumulation in the aorta, leading to mitochondrial reactive oxygen species (ROS) damage and inflammatory responses via excessive mTOR activation (100). Recent studies have further demonstrated that BCAAs metabolic defects may result in local leucine accumulation, which promotes α-cell proliferation through the mTOR pathway (101). Activation of the BCAAs/mTORC1 axis is also associated with insulin sensitivity (11, 102, 103). Mitochondrial BCAAs aminotransferase (BCATm) markedly elevates plasma BCAAs levels, but is not affected by high-fat diet–induced obesity and IR (104, 105), suggesting that BCAAs-mediated effects such as mTOR activation alone are insufficient to induce IR.

Furthermore, activation of mTORC1 stabilizes Grb10, reducing its degradation. Grb10 inhibits the interaction between insulin receptor substrates (IRS) and the insulin receptor via tyrosine phosphorylation, thereby impairing insulin signaling. Studies have shown that eight weeks of treadmill training in T2DM rats effectively reduced elevated Grb10 levels in the hippocampus, restored the mTOR/AMPK signaling pathway, alleviated spatial learning and memory deficits, and enhanced GLUT4 translocation, ultimately improving diabetes-associated cognitive impairment (106). Moreover, evidence shows that β-cell mass and function are increased in diabetic mice with β-cell–specific Grb10 knockout (107), and transcriptome analysis of diabetic mouse liver revealed significant downregulation of Grb10 expression (108). It has also been reported that the food additive carrageenan can impair insulin signaling through two mechanisms: on the one hand, inducing inflammation that elevates Ser(P)307-IRS1 and suppresses insulin signaling; on the other hand, enhancing the GRB10 promoter, whereby GRB10 inhibits Tyr(P)-IRS1, further reducing Ser(P)473-AKT activation. Together, these changes alter IRS1 phosphorylation status and insulin sensitivity (109). Therefore, BCAAs may activate mTORC1 by increasing Grb10 expression, disrupt insulin signaling pathways, and ultimately lead to IR.

Overactivation of the mTORC1 signaling pathway is considered a key mechanism in this process. mTORC1 plays a central role in regulating cell growth and metabolism, while simultaneously inhibiting insulin signaling, thereby affecting glucose uptake and utilization. This inhibitory effect reduces cellular insulin responsiveness and promotes the development of IR. Consequently, modulation of mTORC1 activity may provide a potential therapeutic strategy for diabetes.

## 7 Strategies for reducing IR in T2DM by modulating BCAAs metabolism

BCAAs are closely associated with T2DM and IR, making them a promising therapeutic target for alleviating these conditions. In the field of BCAAs-targeted drug development, several potential candidates have attracted research attention. One class of drugs comprises BCAAs synthesis inhibitors, such as BT2 (3,6-dichloro-2-carboxybenzo[b]thiophene) (110), which block endogenous BCAAs synthesis, thereby reducing circulating levels. Another class of drugs aims to lower BCAAs levels by promoting their oxidation and clearance, including NaPB (28) and fibrate compounds (111), which reduce plasma BCAAs concentrations by enhancing their metabolism and excretion.

### 7.1 BT2

BT2 is a commonly used preclinical inhibitor of BCKDK. BCKDK is a key negative regulator of the BCKDH complex, as it phosphorylates the E1α subunit of BCKDH, leading to enzymatic inactivation and suppression of BCAA oxidation. BT2 promotes BCAA oxidation by inhibiting BCKDK activity, thereby preventing BCKDH phosphorylation and maintaining the complex in an active, dephosphorylated state, which helps restore the homeostasis of BCAAs and BCKAs (112–114). Studies have shown that BT2 inhibits BCKDK, leading to dephosphorylation of the E1 α subunit of BCKD complex, reactivating BCAAs catabolism (100) and improving IR (115). Additionally, research indicates that BT2 can significantly enhance BCAAs catabolism in the colonic tissue of ulcerative colitis (UC) mice by suppressing mTORC1 activation and cyclooxygenase-2 (COX-2) expression (116–118). These findings suggest that dysfunction of BCAAs catabolism plays a key role in the progression of metabolic diseases.

Metformin is a first-line therapeutic agent for patients with T2DM (119). I Studies have shown that during the treatment of obese mice, metformin inhibits BCAAs catabolism, leading to elevated circulating BCAAs levels and thereby, to some extent, limiting its own therapeutic efficacy in the treatment of T2DM. In contrast, BT2 enhances BCAAs catabolism, significantly potentiates the glucose-lowering effect of metformin, and reduces circulating BCAAs levels in ob/ob mice and diet-induced obese (DIO) mice (120). Similarly, dietary restriction of BCAAs intake produced comparable effects. In summary, BT2 is a pharmacological agent capable of directly modulating BCAAs catabolism via activation of BCKD complex. It effectively restores BCAAs catabolism across multiple tissues, alleviates defects in BCAAs catabolic pathways, improves insulin sensitivity, and can synergize with other drugs to enhance glucose-lowering efficacy.

However, recent studies have revealed that, in addition to inhibiting BCKDK and promoting BCAAs oxidation, BT2 also exhibits significant off-target effects: it strongly binds to plasma albumin, displacing albumin-bound tryptophan, thereby markedly reducing plasma tryptophan levels and promoting its degradation into kynurenine, a process independent of classical tryptophan-metabolizing enzymes (121). This finding suggests that while BT2 regulates BCAAs metabolism, it may simultaneously interfere with the tryptophan–kynurenine pathway, raising potential safety concerns. Therefore, although BT2 has demonstrated beneficial effects in multiple animal models, including the improvement of IR, recovery from heart failure, and anti-tumor activities, its potential adverse effects have prevented it from entering clinical application to date (121).

### 7.2 NaPB

NaPB has attracted widespread attention in the biomedical field due to its potential role in BCAAs regulation. Its unique property lies in its ability to lower systemic BCAAs levels, alleviate endoplasmic reticulum (ER) stress, and improve BCAAs-mediated IR (122, 123). Studies have demonstrated that NaPB effectively reduces BCAAs levels (124), and lowering BCAAs concentrations has significant potential to enhance insulin sensitivity and mitigate IR, which is particularly relevant in the treatment of T2DM. In a randomized controlled trial involving T2DM patients, fasting blood samples were collected after oral administration of NaPB or placebo to measure BCAAs, their metabolic intermediates, insulin, triglycerides, free fatty acids (FFA), and glucose levels. Compared to placebo, NaPB increased peripheral insulin sensitivity by 27%, improved mitochondrial oxidation capacity driven by pyruvate in skeletal muscle, enhanced whole-body carbohydrate oxidation under insulin stimulation, and reduced plasma BCAAs and glucose levels, and no adverse events were reported (28).

The mechanism by which NaPB lowers BCAAs levels is attributed to its ability to enhance BCAAs catabolism, thereby accelerating BCAAs degradation and reducing circulating concentrations. NaPB inhibits kinase activity by binding to the specific allosteric pocket of BCKDK, leading to reduced BCAAs levels (114). Abnormally elevated BCAAs levels are closely associated with IR and multiple metabolic disorders, highlighting the significance of NaPB in BCAAs regulation.

In a study involving overweight or obese men, although circulating plasma BCAAs levels were not measured, the results suggested that NaPB may partially ameliorate lipid-induced IR (125). To better understand the regulatory effects of NaPB on human BCAAs catabolism and its metabolic consequences, it is crucial to investigate its impact on insulin signaling and glucose uptake in primary human skeletal muscle cells. Therefore, elucidating the precise molecular mechanisms by which NaPB lowers BCAAs levels, and determining optimal dosing and treatment duration, are of significant importance for future therapeutic applications. In the context of T2DM research, exploring the potential of NaPB holds substantial value.

### 7.3 Targeting the gut microbiota

In recent years, targeting the gut microbiota to regulate metabolic diseases has become a research hotspot. Increasing evidence indicates that the gut microbiota not only participates in host energy metabolism and inflammation regulation but is also closely associated with BCAAs levels. Abnormal BCAAs levels, in turn, are closely linked to IR and T2DM as well as other metabolic disorders. In addition, clinical studies have also suggested that holistic gut microbiota interventions can improve IR. For example, a recent study found that FMT (fecal microbiota transplantation), whether used alone or in combination with metformin, significantly improved HOMA-IR and body mass index in patients with T2DM. Moreover, by promoting donor microbiota engraftment, FMT reshaped the recipients’ gut microbial diversity, thereby facilitating metabolic improvement (126).

In both mouse and human studies, multiple bacterial genera and their related interventions have been shown to regulate BCAAs levels. Notably, in different disease models, the gut microbiota plays a key role in modulating BCAAs metabolism. *Parabacteroides merdae* can promote BCAAs catabolism via its porA gene, thereby alleviating atherosclerosis (127). *Clostridium butyricum* (C.B), as a typical short-chain fatty acid (SCFA)-producing bacterium, can not only lower plasma BCAAs levels in high-fat diet-fed mice by suppressing intestinal BCAAs-producing microbiota, but also ameliorate obesity-related metabolic abnormalities induced thereby, including impaired glucose tolerance, dyslipidemia, and systemic inflammation. Thus, it plays an important role in restoring BCAAs catabolism and alleviating IR (128). *Faecalibacterium prausnitzii* is capable of absorbing and consuming serum BCAAs (57). *Bacteroides ovatus*, under the influence of polymethoxylated flavone-enriched extracts (PMFE), can lower BCAAs levels and improve metabolic syndrome (129). Conversely, certain bacterial genera or environmental factors may elevate BCAAs levels. *Staphylococcus aureus* increases BCAAs via acetolactate synthase (ALS), promoting T2DM development (55); *Bacteroides vulgatus*, regulated by bergenin, increases BCAAs accumulation and exacerbates ulcerative colitis (130); triclosan (TCS) raises plasma BCAAs levels by increasing the *Firmicutes/Bacteroidetes* ratio, thereby impairing glucose tolerance (99). In addition, *Prevotella copri* has BCAAs metabolic capabilities and may contribute to BCAAs levels by producing them in the gut or promoting their absorption (56).

Some natural extracts and pharmacological agents have also been shown experimentally to target gut microbiota abundance, reducing BCAAs levels and improving metabolic outcomes. For example, mulberry leaf aqueous extracts and pimobendan can modulate gut microbial composition to lower BCAAs levels, serving as interventions for T2DM and diabetic cardiomyopathy (58) (**Table 1**).

**Table 1:** Mechanisms by which different gut microbiota target BCAAs.

Microbiome composition	Targeted microbiota drug	Mechanism	Model	Organism	Reference
*Gut Parabacteroides merdae*	\	BCAAs catabolism mediated by the porA gene in P. merdae	Atherosclerosis	Mouse	(127)
*Staphylococcus aureus*	\	Acetolactate synthase (ALS) leads to elevated BCAAs levels	T2DM	Human, Mouse	(55)
*Prevotella copri*	\	Positively correlated with BCAAs metabolism	Sarcopenia	Human, Mouse	(56)
*Clostridium butyricum (C.B)*	\	Suppression of BCAAs-producing bacterial populations	High-fat diet-induced obesity	Mouse	(128)
*Bacteroides vulgatus*	Bergenin	Promotes BCAAs production/accumulation	Ulcerative colitis	Mouse	(130)
*Firmicutes/Bacteroidetes ratio*	Triclosan (TCS)	TCS increases *Firmicutes/Bacteroidetes* ratio, leading to elevated BCAAs levels	Impaired glucose tolerance	Rat	(99)
*Faecalibacterium prausnitzii*	\	Absorbs/consumes serum BCAAs	\	Human	(57)
*Bacteroides ovatus*	Polymethoxylated flavone-enriched extracts (PMFE)	Reduces BCAAs levels	Metabolic syndrome (MetS)	Mouse	(129)
\	Mulberry leaf water extract	Alters gut microbiota, reduces BCAAs	T2DM	Mouse	(58)
\	Pyridostigmine	Alters gut microbiota, reduces BCAAs	Diabetic cardiomyopathy	Mouse	(59)

This table summarizes the major gut microbial genera reported in current studies that target BCAAs metabolism, along with their mechanisms of action.

## 8 Effects of restricting dietary BCAAs intake

studies have shown that dietary restriction of BCAAs can enhance insulin sensitivity through multiple mechanisms and may play a role in diabetes management (131). In an obese mouse model, dietary BCAAs restriction without altering total protein or energy intake resulted in reduced body weight and improved insulin sensitivity and glucose tolerance (132). Another study found that feeding obese mice a diet selectively low in BCAAs improved metabolic health, enhanced glucose tolerance, and reduced fat accumulation (80). These findings indicate that BCAAs restriction can improve insulin sensitivity through multiple mechanisms, thereby alleviating IR (133).

In a 4-week isocaloric intervention with protein intake fixed at 1 g/kg body weight, 12 patients with T2DM consumed diets in which approximately 60% of the protein was provided as an amino acid mixture during weeks 2 and 4. The intervention group received a BCAAs-depleted formula, whereas the control group received a complete amino acid formula. BCAAs restriction reduced fasting, clamp, and postprandial BCAAs levels by 17%, 13%, and 62%, respectively, and was associated with a 24% increase in the oral glucose sensitivity index, a 28% reduction in postprandial insulin secretion, and a 21% increase in circulating Fibroblast Growth Factor 21 (FGF21) levels. In addition, BCAAs restriction was accompanied by downregulation of mTOR signaling in white adipose tissue, an increased mitochondrial respiratory control ratio, and alterations in gut microbiota composition, characterized by an increase in Bacteroidetes and a decrease in Firmicutes. However, under hyperinsulinemic–euglycemic clamp conditions, no significant changes in whole-body or hepatic insulin sensitivity were observed (134).

it is important to note that numerous studies have revealed a close association between elevated BCAAs levels and IR, providing mechanistic support for the theory that “lowering BCAAs levels may improve metabolic disorders.” Nevertheless, at the intervention level, there is currently a lack of direct evidence demonstrating that simply reducing dietary BCAAs intake can reverse IR. Since BCAAs are essential amino acids, excessive restriction may lead to nutritional imbalance, making it a pressing challenge to “meet physiological needs while avoiding excessive accumulation.” Therefore, future research should explore alternative strategies, such as modulating the gut microbiota or developing small-molecule drugs that selectively inhibit BCAAs synthesis or metabolism, to achieve more precise regulation of BCAAs levels.

## 9 Conclusion

IR is one of the central features of T2DM and is closely associated with BCAAs metabolic dysregulation. Increasing evidence indicates that impaired BCAAs metabolism is tightly linked to the metabolic abnormalities and IR observed in T2DM. As essential amino acids, BCAAs play critical roles in protein synthesis and energy metabolism. Multiple studies consistently demonstrate that elevated circulating BCAAs levels are closely associated with the development and progression of IR in T2DM (135–137) supporting the involvement of BCAAs metabolic dysregulation in T2DM pathogenesis.

Moreover, a brief review of BCAAs metabolic pathways reveals that BCAAs catabolism is mediated by multiple enzymes and transporters, generating various intermediates. These intermediates have been shown to regulate key pathways related to glucose and lipid homeostasis as well as insulin sensitivity. Notably, BCAAs not only play a pivotal role in the pathogenesis of T2DM but also represent potential therapeutic targets. Modulating BCAAs metabolism and its associated pathways may improve IR and glycemic control in T2DM patients. Furthermore, elucidating the precise molecular mechanisms by which BCAAs influence IR could facilitate the development of novel interventions and personalized therapeutic strategies, enhancing T2DM management.

In summary, BCAAs metabolic dysregulation is closely associated with metabolic dysfunction and IR in T2DM. A deeper understanding of the complex relationship between BCAAs and IR will provide valuable insights into T2DM pathophysiology and may open new avenues for the treatment of this prevalent metabolic disorder.

## References

[1] Zhai L Wu J Lam YY Kwan HY Bian ZX Wong HLX . Gut-microbial metabolites, probiotics and their roles in type 2 diabetes. Int J Mol Sci. (2021) 22. doi: 10.3390/ijms222312846, PMID: 34884651 PMC8658018

[2] Lee SH Park SY Choi CS . Insulin Resistance: From Mechanisms to Therapeutic Strategies. Diabetes Metab J. (2022) 46:15–37. doi: 10.4093/dmj.2021.0280, PMID: 34965646 PMC8831809

[3] Worldwide trends in diabetes prevalence and treatment from 1990 to 2022: a pooled analysis of 1108 population-representative studies with 141 million participants. Lancet. (2024) 404:2077–93. doi: 10.1016/s0140-6736(24)02317-1, PMID: 39549716 PMC7616842

[4] Cuny T Guerci B Cariou B . New avenues for the pharmacological management of type 2 diabetes: an update. Ann Endocrinol (Paris). (2012) 73:459–68. doi: 10.1016/j.ando.2012.09.002, PMID: 23078974

[5] Kern M Klöting N Mark M Mayoux E Klein T Blüher M . The SGLT2 inhibitor empagliflozin improves insulin sensitivity in db/db mice both as monotherapy and in combination with linagliptin. Metabolism. (2016) 65:114–23. doi: 10.1016/j.metabol.2015.10.010, PMID: 26773934

[6] Fu EL Patorno E Everett BM Vaduganathan M Solomon SD Levin R . Sodium-glucose cotransporter 2 inhibitors vs. sitagliptin in heart failure and type 2 diabetes: an observational cohort study. Eur Heart J. (2023) 44:2216–30. doi: 10.1093/eurheartj/ehad273, PMID: 37259575 PMC10290872

[7] Merzeban DH El Amin Ali AM Hammad RO Elmahdi MH Sofi MA Mahmoud RH . Differential effects of liraglutide naltrexone/bupropion, and caloric restriction on metabolic parameters and beta-cell regeneration in type 2 diabetic rat model: role of beta arrestin 1. J Mol Histol. (2024) 56:50. doi: 10.1007/s10735-024-10326-x, PMID: 39704859

[8] Wang RR Qiu X Pan R Fu H Zhang Z Wang Q . Dietary intervention preserves β cell function in mice through CTCF-mediated transcriptional reprogramming. J Exp Med. (2022) 219. doi: 10.1084/jem.20211779, PMID: 35652891 PMC9166293

[9] Sampath Kumar A Maiya AG Shastry BA Vaishali K Ravishankar N Hazari A . Exercise and insulin resistance in type 2 diabetes mellitus: A systematic review and meta-analysis. Ann Phys Rehabil Med. (2019) 62:98–103. doi: 10.1016/j.rehab.2018.11.001, PMID: 30553010

[10] White PJ McGarrah RW Herman MA Bain JR Shah SH Newgard CB . Insulin action, type 2 diabetes, and branched-chain amino acids: A two-way street. Mol Metab. (2021) 52:101261. doi: 10.1016/j.molmet.2021.101261, PMID: 34044180 PMC8513145

[11] Lynch CJ Adams SH . Branched-chain amino acids in metabolic signaling and insulin resistance. Nat Rev Endocrinol. (2014) 10:723–36. doi: 10.1038/nrendo.2014.171, PMID: 25287287 PMC4424797

[12] Gojda J Cahova M . Gut microbiota as the link between elevated BCAA serum levels and insulin resistance. Biomolecules. (2021) 11. doi: 10.3390/biom11101414, PMID: 34680047 PMC8533624

[13] Himsworth HP . Diabetes mellitus: its differentiation into insulin-sensitive and insulin-insensitive types. 1936. Int J Epidemiol. (2013) 42:1594–8. doi: 10.1093/ije/dyt203, PMID: 24415598

[14] Kahn BB Flier JS . Obesity and insulin resistance. J Clin Invest. (2000) 106:473–81. doi: 10.1172/JCI10842, PMID: 10953022 PMC380258

[15] Kahn CR . Insulin resistance, insulin insensitivity, and insulin unresponsiveness: a necessary distinction. Metabolism. (1978) 27:1893–902. doi: 10.1016/S0026-0495(78)80007-9, PMID: 723640

[16] Kahn SE Hull RL Utzschneider KM . Mechanisms linking obesity to insulin resistance and type 2 diabetes. Nature. (2006) 444:840–6. doi: 10.1038/nature05482, PMID: 17167471

[17] Olefsky JM Revers RR Prince M Henry RR Garvey WT Scarlett JA . Insulin resistance in non-insulin dependent (type II) and insulin dependent (type I) diabetes mellitus. Adv Exp Med Biol. (1985) 189:176–205. 3898763

[18] Reaven GM . Banting lecture 1988. Role of insulin resistance in human disease. Diabetes. (1988) 37:1595–607. doi: 10.2337/diab.37.12.1595, PMID: 3056758

[19] Petersen MC Shulman GI . Mechanisms of insulin action and insulin resistance. Physiol Rev. (2018) 98:2133–223. doi: 10.1152/physrev.00063.2017, PMID: 30067154 PMC6170977

[20] Roden M Shulman GI . The integrative biology of type 2 diabetes. Nature. (2019) 576:51–60. doi: 10.1038/s41586-019-1797-8, PMID: 31802013

[21] Haqq AM Lien LF Boan J Arlotto M Slentz CA Muehlbauer MJ . The Study of the Effects of Diet on Metabolism and Nutrition (STEDMAN) weight loss project: Rationale and design. Contemp Clin Trials. (2005) 26:616–25. doi: 10.1016/j.cct.2005.09.003, PMID: 16239128

[22] Newgard CB An J Bain JR Muehlbauer MJ Stevens RD Lien LF . A branched-chain amino acid-related metabolic signature that differentiates obese and lean humans and contributes to insulin resistance. Cell Metab. (2009) 9:311–26. doi: 10.1016/j.cmet.2009.02.002, PMID: 19356713 PMC3640280

[23] Lee J Vijayakumar A White PJ Xu Y Ilkayeva O Lynch CJ . BCAA supplementation in mice with diet-induced obesity alters the metabolome without impairing glucose homeostasis. Endocrinology. (2021) 162. doi: 10.1210/endocr/bqab062, PMID: 33765118 PMC8183497

[24] Floegel A Stefan N Yu Z Mühlenbruch K Drogan D Joost HG . Identification of serum metabolites associated with risk of type 2 diabetes using a targeted metabolomic approach. Diabetes. (2013) 62:639–48. doi: 10.2337/db12-0495, PMID: 23043162 PMC3554384

[25] Hayashishita A Watanabe T Suzuki N Nakaya T Sugimoto A Yokota I . Insulin resistance assessed by short insulin tolerance test and its association with obesity and insulin resistance-related parameters in humans: A pilot randomized trial. PloS One. (2024) 19:e0297718. doi: 10.1371/journal.pone.0297718, PMID: 38905235 PMC11192359

[26] Tuccinardi D Perakakis N Farr OM Upadhyay J Mantzoros CS . Branched-Chain Amino Acids in relation to food preferences and insulin resistance in obese subjects consuming walnuts: A cross-over, randomized, double-blind, placebo-controlled inpatient physiology study. Clin Nutr. (2021) 40:3032–6. doi: 10.1016/j.clnu.2021.01.020, PMID: 33541836 PMC8172419

[27] Shah H Gannaban RB Haque ZF Dehghani F Kramer A Bowers F . BCAAs acutely drive glucose dysregulation and insulin resistance: role of AgRP neurons. Nutr Diabetes. (2024) 14:40. doi: 10.1038/s41387-024-00298-y, PMID: 38844453 PMC11156648

[28] Vanweert F Neinast M Tapia EE van de Weijer T Hoeks J Schrauwen-Hinderling VB . A randomized placebo-controlled clinical trial for pharmacological activation of BCAA catabolism in patients with type 2 diabetes. Nat Commun. (2022) 13:3508. doi: 10.1038/s41467-022-31249-9, PMID: 35717342 PMC9206682

[29] Sangwung P Petersen KF Shulman GI Knowles JW . Mitochondrial dysfunction, insulin resistance, and potential genetic implications. Endocrinology. (2020) 161. doi: 10.1210/endocr/bqaa017, PMID: 32060542 PMC7341556

[30] Vanweert F de Ligt M Hoeks J Hesselink MKC Schrauwen P Phielix E . Elevated plasma branched-chain amino acid levels correlate with type 2 diabetes-related metabolic disturbances. J Clin Endocrinol Metab. (2021) 106:e1827–36. doi: 10.1210/clinem/dgaa751, PMID: 33079174

[31] Mthembu SXH Dludla PV Nyambuya TM Kappo AP Madoroba E Ziqubu K . Experimental models of lipid overload and their relevance in understanding skeletal muscle insulin resistance and pathological changes in mitochondrial oxidative capacity. Biochimie. (2022) 196:182–93. doi: 10.1016/j.biochi.2021.09.010, PMID: 34563603

[32] Jang C Oh SF Wada S Rowe GC Liu L Chan MC . A branched-chain amino acid metabolite drives vascular fatty acid transport and causes insulin resistance. Nat Med. (2016) 22:421–6. doi: 10.1038/nm.4057, PMID: 26950361 PMC4949205

[33] Yoon MS . The emerging role of branched-chain amino acids in insulin resistance and metabolism. Nutrients. (2016) 8. doi: 10.3390/nu8070405, PMID: 27376324 PMC4963881

[34] Stipanuk MH . Leucine and protein synthesis: mTOR and beyond. Nutr Rev. (2007) 65:122–9. doi: 10.1111/j.1753-4887.2007.tb00289.x, PMID: 17425063

[35] Smith GI Yoshino J Stromsdorfer KL Klein SJ Magkos F Reeds DN . Protein ingestion induces muscle insulin resistance independent of leucine-mediated mTOR activation. Diabetes. (2015) 64:1555–63. doi: 10.2337/db14-1279, PMID: 25475435 PMC4407849

[36] Benedini S Ermetici F Briganti S Codella R Terruzzi I Maffi P . Insulin-mimetic effects of short-term rapamycin in type 1 diabetic patients prior to islet transplantation. Acta Diabetol. (2018) 55:715–22. doi: 10.1007/s00592-018-1141-z, PMID: 29654388

[37] Hay N Sonenberg N . Upstream and downstream of mTOR. Genes Dev. (2004) 18:1926–45. doi: 10.1101/gad.1212704, PMID: 15314020

[38] Um SH D’Alessio D Thomas G . Nutrient overload, insulin resistance, and ribosomal protein S6 kinase 1, S6K1. Cell Metab. (2006) 3:393–402. doi: 10.1016/j.cmet.2006.05.003, PMID: 16753575

[39] Um SH Frigerio F Watanabe M Picard F Joaquin M Sticker M . Absence of S6K1 protects against age- and diet-induced obesity while enhancing insulin sensitivity. Nature. (2004) 431:200–5. doi: 10.1038/nature02866, PMID: 15306821

[40] Vanweert F Schrauwen P Phielix E . Role of branched-chain amino acid metabolism in the pathogenesis of obesity and type 2 diabetes-related metabolic disturbances BCAA metabolism in type 2 diabetes. Nutr Diabetes. (2022) 12:35. doi: 10.1038/s41387-022-00213-3, PMID: 35931683 PMC9356071

[41] Grimble GK . Mechanisms of peptide and amino acid transport and their regulation. Nestle Nutr Workshop Ser Clin Perform Program. (2000) 3:63–84; discussion 84-8. doi: 10.1159/000061797, PMID: 11490614

[42] Bröer S Bröer A . Amino acid homeostasis and signaling in mammalian cells and organisms. Biochem J. (2017) 474:1935–63. doi: 10.1042/bcj20160822, PMID: 28546457 PMC5444488

[43] Ichihara A . Isozyme patterns of branched-chain amino acid transaminase during cellular differentiation and carcinogenesis. Ann N Y Acad Sci. (1975) 259:347–54. doi: 10.1111/j.1749-6632.1975.tb25431.x, PMID: 54031

[44] Holeček M . Branched-chain amino acids and branched-chain keto acids in hyperammonemic states: metabolism and as supplements. Metabolites. (2020) 10. doi: 10.3390/metabo10080324, PMID: 32784821 PMC7464849

[45] Adeva-Andany MM López-Maside L Donapetry-García C Fernández-Fernández C Sixto-Leal C . Enzymes involved in branched-chain amino acid metabolism in humans. Amino Acids. (2017) 49:1005–28. doi: 10.1007/s00726-017-2412-7, PMID: 28324172

[46] Wynn RM Kato M Machius M Chuang JL Li J Tomchick DR . Molecular mechanism for regulation of the human mitochondrial branched-chain alpha-ketoacid dehydrogenase complex by phosphorylation. Structure. (2004) 12:2185–96. doi: 10.1016/j.str.2004.09.013, PMID: 15576032

[47] Neinast MD Jang C Hui S Murashige DS Chu Q Morscher RJ . Quantitative analysis of the whole-body metabolic fate of branched-chain amino acids. Cell Metab. (2019) 29:417–429.e4. doi: 10.1016/j.cmet.2018.10.013, PMID: 30449684 PMC6365191

[48] Newton-Tanzer E Can SN Demmelmair H Horak J Holdt L Koletzko B . Apparent saturation of branched-chain amino acid catabolism after high dietary milk protein intake in healthy adults. J Clin Endocrinol Metab. (2025) 110:e1793–801. doi: 10.1210/clinem/dgae599, PMID: 39302872 PMC12086401

[49] Prodhan UK Milan AM Thorstensen EB Barnett MPG Stewart RAH Benatar JR . Altered dairy protein intake does not alter circulatory branched chain amino acids in healthy adults: A randomized controlled trial. Nutrients. (2018) 10. doi: 10.3390/nu10101510, PMID: 30326639 PMC6213722

[50] Wang Y Zhao X Ma Y Yang Y Ge S . The effects of vitamin B6 on the nutritional support of BCAAs-enriched amino acids formula in rats with partial gastrectomy. Clin Nutr. (2023) 42:954–61. doi: 10.1016/j.clnu.2023.04.018, PMID: 37104913

[51] Van Hul M Cani PD . The gut microbiota in obesity and weight management: microbes as friends or foe? Nat Rev Endocrinol. (2023) 19:258–71. doi: 10.1038/s41574-022-00794-0, PMID: 36650295

[52] Adak A Khan MR . An insight into gut microbiota and its functionalities. Cell Mol Life Sci. (2019) 76:473–93. doi: 10.1007/s00018-018-2943-4, PMID: 30317530 PMC11105460

[53] Schoeler M Caesar R . Dietary lipids, gut microbiota and lipid metabolism. Rev Endocr Metab Disord. (2019) 20:461–72. doi: 10.1007/s11154-019-09512-0, PMID: 31707624 PMC6938793

[54] Lee CJ Sears CL Maruthur N . Gut microbiome and its role in obesity and insulin resistance. Ann N Y Acad Sci. (2020) 1461:37–52. doi: 10.1111/nyas.14107, PMID: 31087391

[55] Liang T . Gut microbiota-driven BCAA biosynthesis via Staphylococcus aureus -expressed acetolactate synthase impairs glycemic control in type 2 diabetes in South China. Microbiol Res. (2025) 296:128145. doi: 10.1016/j.micres.2025.128145, PMID: 40138872

[56] Liu X Wu J Tang J Xu Z Zhou B Liu Y . Prevotella copri alleviates sarcopenia via attenuating muscle mass loss and function decline. J Cachexia Sarcopenia Muscle. (2023) 14:2275–88. doi: 10.1002/jcsm.13313, PMID: 37591518 PMC10570070

[57] Moran-Ramos S Macias-Kauffer L López-Contreras BE Villamil-Ramírez H Ocampo-Medina E León-Mimila P . A higher bacterial inward BCAA transport driven by Faecalibacterium prausnitzii is associated with lower serum levels of BCAA in early adolescents. Mol Med. (2021) 27:108. doi: 10.1186/s10020-021-00371-7, PMID: 34525937 PMC8444488

[58] Zheng XX Li DX Li YT Chen YL Zhao YL Ji S . Mulberry leaf water extract alleviates type 2 diabetes in mice via modulating gut microbiota-host co-metabolism of branched-chain amino acid. Phytother Res. (2023) 37:3195–210. doi: 10.1002/ptr.7822, PMID: 37013717

[59] Yang Y Zhao M He X Wu Q Li DL Zang WJ . Pyridostigmine protects against diabetic cardiomyopathy by regulating vagal activity, gut microbiota, and branched-chain amino acid catabolism in diabetic mice. Front Pharmacol. (2021) 12:647481. doi: 10.3389/fphar.2021.647481, PMID: 34084135 PMC8167056

[60] Patrick M Gu Z Zhang G Wynn RM Kaphle P Cao H . Metabolon formation regulates branched-chain amino acid oxidation and homeostasis. Nat Metab. (2022) 4:1775–91. doi: 10.1038/s42255-022-00689-4, PMID: 36443523 PMC11977170

[61] Uddin GM Karwi QG Pherwani S Gopal K Wagg CS Biswas D . Deletion of BCATm increases insulin-stimulated glucose oxidation in the heart. Metabolism. (2021) 124:154871. doi: 10.1016/j.metabol.2021.154871, PMID: 34478752

[62] Abiri M Saei H Eghbali M Karamzadeh R Shirzadeh T Sharifi Z . Maple syrup urine disease mutation spectrum in a cohort of 40 consanguineous patients and insilico analysis of novel mutations. Metab Brain Dis. (2019) 34:1145–56. doi: 10.1007/s11011-019-00435-y, PMID: 31119508

[63] Strand JM Skinnes R Scheffler K Rootvelt T Woldseth B Bjørås M . Genome instability in Maple Syrup Urine Disease correlates with impaired mitochondrial biogenesis. Metabolism. (2014) 63:1063–70. doi: 10.1016/j.metabol.2014.05.003, PMID: 24928662

[64] Yoshida N Oi Y Kitaura Y Shimomura Y . Activation of hepatic branched-chain α-ketoacid dehydrogenase complex by vitamin D deficiency in rats. J Nutr Sci Vitaminol (Tokyo). (2023) 69:490–2. doi: 10.3177/jnsv.69.490, PMID: 38171823

[65] Roth Flach RJ Bollinger E Reyes AR Laforest B Kormos BL Liu S . Small molecule branched-chain ketoacid dehydrogenase kinase (BDK) inhibitors with opposing effects on BDK protein levels. Nat Commun. (2023) 14:4812. doi: 10.1038/s41467-023-40536-y, PMID: 37558654 PMC10412597

[66] Bollinger E Peloquin M Libera J Albuquerque B Pashos E Shipstone A . BDK inhibition acts as a catabolic switch to mimic fasting and improve metabolism in mice. Mol Metab. (2022) 66:101611. doi: 10.1016/j.molmet.2022.101611, PMID: 36220546 PMC9589198

[67] Suryawan A Hawes JW Harris RA Shimomura Y Jenkins AE Hutson SM . A molecular model of human branched-chain amino acid metabolism. Am J Clin Nutr. (1998) 68:72–81. doi: 10.1093/ajcn/68.1.72, PMID: 9665099

[68] Mu L Ye Z Hu J Zhang Y Chen K Sun H . PPM1K-regulated impaired catabolism of branched-chain amino acids orchestrates polycystic ovary syndrome. EBioMedicine. (2023) 89:104492. doi: 10.1016/j.ebiom.2023.104492, PMID: 36863088 PMC9986518

[69] Ozcelik F Arslan S Ozguc Caliskan B Kardas F Ozkul Y Dundar M . PPM1K defects cause mild maple syrup urine disease: The second case in the literature. Am J Med Genet A. (2023) 191:1360–5. doi: 10.1002/ajmg.a.63129, PMID: 36706222

[70] Sugimoto T Kamei Y . Regulation of skeletal muscle function by amino acids, especially non-proteinogenic amino acids. J Nutr Sci Vitaminol (Tokyo). (2022) 68:S31–s33. doi: 10.3177/jnsv.68.S31, PMID: 36437009

[71] Imai D Nakanishi N Shinagawa N Yamamoto S Ichikawa T Sumi M . Association of elevated serum branched-chain amino acid levels with longitudinal skeletal muscle loss. J Endocr Soc. (2024) 8:bvad178. doi: 10.1210/jendso/bvad178, PMID: 38213909 PMC10783241

[72] Lu KY Primus Dass KT Tsai SF Chuang HM Lin SZ Liu SP . Clinical application potential of small molecules that induce brown adipose tissue thermogenesis by improving fat metabolism. Cell Transplant. (2020) 29:963689720927394. doi: 10.1177/0963689720927394, PMID: 32854518 PMC7563884

[73] Whytock KL Divoux A Sun Y Pino MF Yu G Jin CA . Aging human abdominal subcutaneous white adipose tissue at single cell resolution. Aging Cell. (2024) 23:e14287. doi: 10.1111/acel.14287, PMID: 39141531 PMC11561672

[74] Lackey DE Lynch CJ Olson KC Mostaedi R Ali M Smith WH . Regulation of adipose branched-chain amino acid catabolism enzyme expression and cross-adipose amino acid flux in human obesity. Am J Physiol Endocrinol Metab. (2013) 304:E1175–87. doi: 10.1152/ajpendo.00630.2012, PMID: 23512805 PMC3680678

[75] Mardinoglu A Gogg S Lotta LA Stančáková A Nerstedt A Boren J . Elevated plasma levels of 3-hydroxyisobutyric acid are associated with incident type 2 diabetes. EBioMedicine. (2018) 27:151–5. doi: 10.1016/j.ebiom.2017.12.008, PMID: 29246479 PMC5828558

[76] Ma QX Zhu WY Lu XC Jiang D Xu F Li JT . BCAA-BCKA axis regulates WAT browning through acetylation of PRDM16. Nat Metab. (2022) 4:106–22. doi: 10.1038/s42255-021-00520-6, PMID: 35075301

[77] Tărlungeanu DC Deliu E Dotter CP Kara M Janiesch PC Scalise M . Impaired amino acid transport at the blood brain barrier is a cause of autism spectrum disorder. Cell. (2016) 167:1481–1494.e18. doi: 10.1016/j.cell.2016.11.013, PMID: 27912058 PMC5554935

[78] Fernstrom JD . Branched-chain amino acids and brain function. J Nutr. (2005) 135:1539s–46s. doi: 10.1093/jn/135.6.1539S, PMID: 15930466

[79] Yudkoff M . Brain metabolism of branched-chain amino acids. Glia. (1997) 21:92–8. doi: 10.1002/(SICI)1098-1136(199709)21:1<92::AID-GLIA10>3.0.CO;2-W 9298851

[80] Fontana L Cummings NE Arriola Apelo SI Neuman JC Kasza I Schmidt BA . Decreased consumption of branched-chain amino acids improves metabolic health. Cell Rep. (2016) 16:520–30. doi: 10.1016/j.celrep.2016.05.092, PMID: 27346343 PMC4947548

[81] Yu D Richardson NE Green CL Spicer AB Murphy ME Flores V . The adverse metabolic effects of branched-chain amino acids are mediated by isoleucine and valine. Cell Metab. (2021) 33:905–922.e6. doi: 10.1016/j.cmet.2021.03.025, PMID: 33887198 PMC8102360

[82] Huang H Chen H Yao Y Lou X . Branched-chain amino acids supplementation induces insulin resistance and pro-inflammatory macrophage polarization via INFGR1/JAK1/STAT1 signal pathway. Mol Med. (2024) 30:149. doi: 10.1186/s10020-024-00894-9, PMID: 39267003 PMC11391606

[83] Mita MM Mita A Rowinsky EK . Mammalian target of rapamycin: a new molecular target for breast cancer. Clin Breast Cancer. (2003) 4:126–37. doi: 10.3816/CBC.2003.n.018, PMID: 12864941

[84] Marafie SK Al-Mulla F Abubaker J . mTOR: its critical role in metabolic diseases, cancer, and the aging process. Int J Mol Sci. (2024) 25. doi: 10.3390/ijms25116141, PMID: 38892329 PMC11173325

[85] Yang M Lu Y Piao W Jin H . The translational regulation in mTOR pathway. Biomolecules. (2022) 12. doi: 10.3390/biom12060802, PMID: 35740927 PMC9221026

[86] Frappaolo A Giansanti MG . Using Drosophila melanogaster to Dissect the Roles of the mTOR Signaling Pathway in Cell Growth. Cells. (2023) 12. doi: 10.3390/cells12222622, PMID: 37998357 PMC10670727

[87] Vary TC Deiter G Lynch CJ . Rapamycin limits formation of active eukaryotic initiation factor 4F complex following meal feeding in rat hearts. J Nutr. (2007) 137:1857–62. doi: 10.1093/jn/137.8.1857, PMID: 17634255

[88] Vary TC Anthony JC Jefferson LS Kimball SR Lynch CJ . Rapamycin blunts nutrient stimulation of eIF4G, but not PKCepsilon phosphorylation, in skeletal muscle. Am J Physiol Endocrinol Metab. (2007) 293:E188–96. doi: 10.1152/ajpendo.00037.2007, PMID: 17389711

[89] Vary TC Lynch CJ . Meal feeding enhances formation of eIF4F in skeletal muscle: role of increased eIF4E availability and eIF4G phosphorylation. Am J Physiol Endocrinol Metab. (2006) 290:E631–42. doi: 10.1152/ajpendo.00460.2005, PMID: 16263769

[90] Wang X Proud CG . The mTOR pathway in the control of protein synthesis. Physiol (Bethesda). (2006) 21:362–9. doi: 10.1152/physiol.00024.2006, PMID: 16990457

[91] Saha AK Xu XJ Lawson E Deoliveira R Brandon AE Kraegen EW . Downregulation of AMPK accompanies leucine- and glucose-induced increases in protein synthesis and insulin resistance in rat skeletal muscle. Diabetes. (2010) 59:2426–34. doi: 10.2337/db09-1870, PMID: 20682696 PMC3279521

[92] Mokbel N Hoffman NJ Girgis CM Small L Turner N Daly RJ . Grb10 deletion enhances muscle cell proliferation, differentiation and GLUT4 plasma membrane translocation. J Cell Physiol. (2014) 229:1753–64. doi: 10.1002/jcp.24628, PMID: 24664951

[93] Calejman CM Doxsey WG Fazakerley DJ Guertin DA . Integrating adipocyte insulin signaling and metabolism in the multi-omics era. Trends Biochem Sci. (2022) 47:531–46. doi: 10.1016/j.tibs.2022.02.009, PMID: 35304047 PMC9109456

[94] Cuomo P Capparelli R Iannelli A Iannelli D . Role of branched-chain amino acid metabolism in type 2 diabetes, obesity, cardiovascular disease and non-alcoholic fatty liver disease. Int J Mol Sci. (2022) 23. doi: 10.3390/ijms23084325, PMID: 35457142 PMC9030262

[95] Du W Jiang S Yin S Wang R Zhang C Yin BC . The microbiota-dependent tryptophan metabolite alleviates high-fat diet-induced insulin resistance through the hepatic AhR/TSC2/mTORC1 axis. Proc Natl Acad Sci U.S.A. (2024) 121:e2400385121. doi: 10.1073/pnas.2400385121, PMID: 39167602 PMC11363250

[96] Tremblay F Brûlé S Um SH Li Y Masuda K Roden M . Identification of IRS-1 Ser-1101 as a target of S6K1 in nutrient- and obesity-induced insulin resistance. Proc Natl Acad Sci U.S.A. (2007) 104:14056–61. doi: 10.1073/pnas.0706517104, PMID: 17709744 PMC1950339

[97] Hsu PP Kang SA Rameseder J Zhang Y Ottina KA Lim D . The mTOR-regulated phosphoproteome reveals a mechanism of mTORC1-mediated inhibition of growth factor signaling. Science. (2011) 332:1317–22. doi: 10.1126/science.1199498, PMID: 21659604 PMC3177140

[98] Zhang J Gao Z Yin J Quon MJ Ye J . S6K directly phosphorylates IRS-1 on Ser-270 to promote insulin resistance in response to TNF-(alpha) signaling through IKK2. J Biol Chem. (2008) 283:35375–82. doi: 10.1074/jbc.M806480200, PMID: 18952604 PMC2602883

[99] Yu Z Han J Li L Zhang Q Chen A Chen J . Chronic triclosan exposure induce impaired glucose tolerance by altering the gut microbiota. Food Chem Toxicol. (2024) 183:114305. doi: 10.1016/j.fct.2023.114305, PMID: 38052405

[100] Yu L Huang T Zhao J Zhou Z Cao Z Chi Y . Branched-chain amino acid catabolic defect in vascular smooth muscle cells drives thoracic aortic dissection via mTOR hyperactivation. Free Radic Biol Med. (2024) 210:25–41. doi: 10.1016/j.freeradbiomed.2023.11.002, PMID: 37956909

[101] Yang Y Wang S Sheng C Tan J Chen J Li T . Branched-chain amino acid catabolic defect promotes α-cell proliferation via activating mTOR signaling. Mol Cell Endocrinol. (2024) 582:112143. doi: 10.1016/j.mce.2023.112143, PMID: 38158148

[102] Macotela Y Emanuelli B Bång AM Espinoza DO Boucher J Beebe K . Dietary leucine--an environmental modifier of insulin resistance acting on multiple levels of metabolism. PloS One. (2011) 6:e21187. doi: 10.1371/journal.pone.0021187, PMID: 21731668 PMC3120846

[103] Zhang Y Guo K LeBlanc RE Loh D Schwartz GJ Yu YH . Increasing dietary leucine intake reduces diet-induced obesity and improves glucose and cholesterol metabolism in mice via multimechanisms. Diabetes. (2007) 56:1647–54. doi: 10.2337/db07-0123, PMID: 17360978

[104] She P Reid TM Bronson SK Vary TC Hajnal A Lynch CJ . Disruption of BCATm in mice leads to increased energy expenditure associated with the activation of a futile protein turnover cycle. Cell Metab. (2007) 6:181–94. doi: 10.1016/j.cmet.2007.08.003, PMID: 17767905 PMC2693888

[105] Mechchate H Abdualkader AM Bernacchi JB Gopal K Tabatabaei Dakhili SA Yang K . Defective muscle ketone body oxidation disrupts BCAA catabolism by altering mitochondrial branched-chain aminotransferase. Am J Physiol Endocrinol Metab. (2023) 324:E425–e436. doi: 10.1152/ajpendo.00206.2022, PMID: 36989424

[106] Zhang Y Chen D Zhang M Bian J Qian S Kou X . Treadmill training attenuate STZ-induced cognitive dysfunction in type 2 diabetic rats via modulating Grb10/IGF-R signaling. Brain Res Bull. (2022) 181:12–20. doi: 10.1016/j.brainresbull.2022.01.007, PMID: 35065184

[107] Cai Z Liu F Yang Y Li D Hu S Song L . GRB10 regulates β-cell mass by inhibiting β-cell proliferation and stimulating β-cell dedifferentiation. J Genet Genomics. (2022) 49:208–16. doi: 10.1016/j.jgg.2021.11.006, PMID: 34861413

[108] Ge Q Zhang S Chen L Tang M Liu L Kang M . Mulberry leaf regulates differentially expressed genes in diabetic mice liver based on RNA-seq analysis. Front Physiol. (2018) 9:1051. doi: 10.3389/fphys.2018.01051, PMID: 30131712 PMC6090096

[109] Bhattacharyya S Feferman L Tobacman JK . Carrageenan Inhibits Insulin Signaling through GRB10-mediated Decrease in Tyr(P)-IRS1 and through Inflammation-induced Increase in Ser(P)307-IRS1. J Biol Chem. (2015) 290:10764–74. doi: 10.1074/jbc.M114.630053, PMID: 25784556 PMC4409242

[110] Acevedo A Jones AE Danna BT Turner R Montales KP Benincá C . The BCKDK inhibitor BT2 is a chemical uncoupler that lowers mitochondrial ROS production and de novo lipogenesis. J Biol Chem. (2024) 300:105702. doi: 10.1016/j.jbc.2024.105702, PMID: 38301896 PMC10910128

[111] Ishiguro H Jones AE Danna BT Turner R Montales KP Benincá C . Clofibrate treatment promotes branched-chain amino acid catabolism and decreases the phosphorylation state of mTOR, eIF4E-BP1, and S6K1 in rat liver. Life Sci. (2006) 79:737–43. doi: 10.1016/j.lfs.2006.02.037, PMID: 16616211

[112] Voronova V Sokolov V Morias Y Boezelman MJ Wågberg M Henricsson M . Evaluation of therapeutic strategies targeting BCAA catabolism using a systems pharmacology model. Front Pharmacol. (2022) 13:993422. doi: 10.3389/fphar.2022.993422, PMID: 36518669 PMC9744226

[113] Zuo X Zhao R Wu M Wang Y Wang S Tang K . Multi-omic profiling of sarcopenia identifies disrupted branched-chain amino acid catabolism as a causal mechanism and therapeutic target. Nat Aging. (2025) 5:419–36. doi: 10.1038/s43587-024-00797-8, PMID: 39910243

[114] Tso SC Gui WJ Wu CY Chuang JL Qi X Skvora KJ . Benzothiophene carboxylate derivatives as novel allosteric inhibitors of branched-chain α-ketoacid dehydrogenase kinase. J Biol Chem. (2014) 289:20583–93. doi: 10.1074/jbc.M114.569251, PMID: 24895126 PMC4110271

[115] Li T Zhao L Li Y Dang M Lu J Lu Z . PPM1K mediates metabolic disorder of branched-chain amino acid and regulates cerebral ischemia-reperfusion injury by activating ferroptosis in neurons. Cell Death Dis. (2023) 14:634. doi: 10.1038/s41419-023-06135-x, PMID: 37752100 PMC10522625

[116] Sun H Olson KC Gao C Prosdocimo DA Zhou M Wang Z . Catabolic defect of branched-chain amino acids promotes heart failure. Circulation. (2016) 133:2038–49. doi: 10.1161/CIRCULATIONAHA.115.020226, PMID: 27059949 PMC4879058

[117] Wang W Zhang F Xia Y Zhao S Yan W Wang H . Defective branched chain amino acid catabolism contributes to cardiac dysfunction and remodeling following myocardial infarction. Am J Physiol Heart Circ Physiol. (2016) 311:H1160–h1169. doi: 10.1152/ajpheart.00114.2016, PMID: 27542406

[118] Chen M Gao C Yu J Ren S Wang M Wynn RM . Therapeutic effect of targeting branched-chain amino acid catabolic flux in pressure-overload induced heart failure. J Am Heart Assoc. (2019) 8:e011625. doi: 10.1161/JAHA.118.011625, PMID: 31433721 PMC6585363

[119] Sanchez-Rangel E Inzucchi SE . Metformin: clinical use in type 2 diabetes. Diabetologia. (2017) 60:1586–93. doi: 10.1007/s00125-017-4336-x, PMID: 28770321

[120] Zhao X Zhang X Pei J Liu Y Niu W Sun H . Targeting BCAA metabolism to potentiate metformin’s therapeutic efficacy in the treatment of diabetes in mice. Diabetologia. (2023) 66:2139–53. doi: 10.1007/s00125-023-05985-6, PMID: 37581618

[121] Bowman CE Neinast MD Jang C Patel J Blair MC Mirek ET . Off-target depletion of plasma tryptophan by allosteric inhibitors of BCKDK. bioRxiv. (2024). doi: 10.1101/2024.03.05.582974, PMID: 40348014 PMC12149413

[122] Crossland H Smith K Idris I Phillips BE Atherton PJ Wilkinson DJ . Phenylbutyrate, a branched-chain amino acid keto dehydrogenase activator, promotes branched-chain amino acid metabolism and induces muscle catabolism in C2C12 cells. Exp Physiol. (2021) 106:585–92. doi: 10.1113/EP089223, PMID: 33369803 PMC9291829

[123] Rivera CN Smith CE Draper LV Watne RM Wommack AJ Vaughan RA . Physiological 4-phenylbutyrate promotes mitochondrial biogenesis and metabolism in C2C12 myotubes. Biochimie. (2024) 219:155–64. doi: 10.1016/j.biochi.2023.11.009, PMID: 38008282

[124] Osaka S Nakano S Mizuno T Hiraoka Y Minowa K Hirai S . A randomized trial to examine the impact of food on pharmacokinetics of 4-phenylbutyrate and change in amino acid availability after a single oral administration of sodium 4-phenylbutyrarte in healthy volunteers. Mol Genet Metab. (2021) 132:220–6. doi: 10.1016/j.ymgme.2021.02.002, PMID: 33648834

[125] Xiao C Giacca A Lewis GF . Sodium phenylbutyrate, a drug with known capacity to reduce endoplasmic reticulum stress, partially alleviates lipid-induced insulin resistance and beta-cell dysfunction in humans. Diabetes. (2011) 60:918–24. doi: 10.2337/db10-1433, PMID: 21270237 PMC3046853

[126] Wu Z Zhang B Chen F Xia R Zhu D Chen B . Fecal microbiota transplantation reverses insulin resistance in type 2 diabetes: A randomized, controlled, prospective study. Front Cell Infect Microbiol. (2022) 12:1089991. doi: 10.3389/fcimb.2022.1089991, PMID: 36704100 PMC9872724

[127] Qiao S Liu C Sun L Wang T Dai H Wang K . Gut Parabacteroides merdae protects against cardiovascular damage by enhancing branched-chain amino acid catabolism. Nat Metab. (2022) 4:1271–86. doi: 10.1038/s42255-022-00649-y, PMID: 36253620

[128] Zhang X Li Z Cao J Sun H Niu W . Clostridium Butyricum 337279 shapes the gut microbiota to attenuate metabolic disorder in diet-induced obese mice. Front Microbiol. (2025) 16:1580847. doi: 10.3389/fmicb.2025.1580847, PMID: 40415947 PMC12098643

[129] Zeng SL Li SZ Xiao PT Cai YY Chu C Chen BZ . Citrus polymethoxyflavones attenuate metabolic syndrome by regulating gut microbiome and amino acid metabolism. Sci Adv. (2020) 6:eaax6208. doi: 10.1126/sciadv.aax6208, PMID: 31922003 PMC6941918

[130] Huang TQ Chen YX Zeng SL Lin Y Li F Jiang ZM . Bergenin alleviates ulcerative colitis by decreasing gut commensal bacteroides vulgatus-mediated elevated branched-chain amino acids. J Agric Food Chem. (2024) 72:3606–21. doi: 10.1021/acs.jafc.3c09448, PMID: 38324392

[131] Aman Y . Isoleucine dietary restriction boosts healthspan and longevity in mice. Nat Aging. (2023) 3:1471. doi: 10.1038/s43587-023-00547-2, PMID: 38057387

[132] Cummings NE Williams EM Kasza I Konon EN Schaid MD Schmidt BA . Restoration of metabolic health by decreased consumption of branched-chain amino acids. J Physiol. (2018) 596:623–45. doi: 10.1113/jp275075, PMID: 29266268 PMC5813603

[133] Ramzan I Taylor M Phillips B Wilkinson D Smith K Hession K . A novel dietary intervention reduces circulatory branched-chain amino acids by 50%: A pilot study of relevance for obesity and diabetes. Nutrients. (2020) 13. doi: 10.3390/nu13010095, PMID: 33396718 PMC7824725

[134] Karusheva Y Koessler T Strassburger K Markgraf D Mastrototaro L Jelenik T . Short-term dietary reduction of branched-chain amino acids reduces meal-induced insulin secretion and modifies microbiome composition in type 2 diabetes: a randomized controlled crossover trial. Am J Clin Nutr. (2019) 110:1098–107. doi: 10.1093/ajcn/nqz191, PMID: 31667519 PMC6821637

[135] Moreno-Vedia J Llop D Rodríguez-Calvo R Plana N Amigó N Rosales R . Serum branch-chained amino acids are increased in type 2 diabetes and associated with atherosclerotic cardiovascular disease. Cardiovasc Diabetol. (2023) 22:249. doi: 10.1186/s12933-023-01958-6, PMID: 37710233 PMC10503204

[136] Sawicki KT Ning H Allen NB Carnethon MR Wallia A Otvos JD . Longitudinal trajectories of branched chain amino acids through young adulthood and diabetes in later life. JCI Insight. (2024) 9. doi: 10.1172/jci.insight.181901, PMID: 38855872 PMC11382874

[137] Ramzan I Ardavani A Vanweert F Mellett A Atherton PJ Idri I . The association between circulating branched chain amino acids and the temporal risk of developing type 2 diabetes mellitus: A systematic review & Meta-analysis. Nutrients. (2022) 14. doi: 10.3390/nu14204411, PMID: 36297095 PMC9610746

